# Detailed cytogenetic analysis of three duck species (the northern pintail, mallard, and common goldeneye) and karyotype evolution in the family Anatidae (Anseriformes, Aves)

**DOI:** 10.18699/vjgb-24-84

**Published:** 2024-11

**Authors:** V.R. Beklemisheva, K.V. Tishakova, S.A. Romanenko, D.A. Andreushkova, V.A. Yudkin, E.А. Interesova, F. Yang, M.A. Ferguson-Smith, A.S. Graphodatsky, A.A. Proskuryakova

**Affiliations:** Institute of Molecular and Cellular Biology of the Siberian Branch of the Russian Academy of Sciences, Novosibirsk, Russia; Institute of Molecular and Cellular Biology of the Siberian Branch of the Russian Academy of Sciences, Novosibirsk, Russia; Institute of Molecular and Cellular Biology of the Siberian Branch of the Russian Academy of Sciences, Novosibirsk, Russia; Institute of Molecular and Cellular Biology of the Siberian Branch of the Russian Academy of Sciences, Novosibirsk, Russia; Institute of Systematics and Ecology of Animals of the Siberian Branch of the Russian Academy of Sciences, Novosibirsk, Russia Novosibirsk State University, Novosibirsk, Russia; Institute of Systematics and Ecology of Animals of the Siberian Branch of the Russian Academy of Sciences, Novosibirsk, Russia Tomsk State University, Tomsk, Russia; School of Life Sciences and Medicine, Shandong University of Technology, Zibo, China; Cambridge Resource Center for Comparative Genomics, University of Cambridge, Cambridge, United Kingdom; Institute of Molecular and Cellular Biology of the Siberian Branch of the Russian Academy of Sciences, Novosibirsk, Russia; Institute of Molecular and Cellular Biology of the Siberian Branch of the Russian Academy of Sciences, Novosibirsk, Russia

**Keywords:** Anas acuta, Anas platyrhynchos, Bucephala clangula, Burhinus oedicnemus, comparative chromosome painting, constitutive heterochromatin, ribosomal genes, telomere, Anas acuta, Anas platyrhynchos, Bucephala clangula, сравнительный хромосомный пэйнтинг, конститутивный гетерохроматин, рибосомные гены, теломера

## Abstract

Galliformes and Anseriformes are two branches of the Galloanserae group, basal to other Neognathae. In contrast to Galliformes, Anseriformes have not been thoroughly researched by cytogenetic methods. This report is focused on representatives of Anseriformes and the evolution of their chromosome sets. Detailed cytogenetic analysis (G-banding, C- banding, and fluorescence in situ hybridization) was performed on three duck species: the northern pintail (Anas acuta, 2n = 80), the mallard (A. platyrhynchos, 2n = 80), and the common goldeneye (Bucephala clangula, 2n = 80). Using stone curlew (Burhinus oedicnemus, 2n = 42, Charadriiformes) chromosome painting probes, we created homology maps covering macrochromosomes and some microchromosomes. The results indicated a high level of syntenic group conservation among the duck genomes. The two Anas species share their macrochromosome number, whereas in B. clangula, this number is increased due to fissions of two ancestral elements. Additionally, in this species, the presence of massive heterochromatic blocks in most macroautosomes and sex chromosomes was discovered. Localization of clusters of ribosomal DNA and telomere repeats revealed that the duck karyotypes contain some microchromosomes that bear ribosomal RNA genes and/or are enriched for telomere repeats and constitutive heterochromatin. Dot plot (D-GENIES) analysis confirmed the established view about the high level of syntenic group conservation among Anatidae genomes. The new data about the three Anatidae species add knowledge about the transformation of macro- and sex chromosomes of Anseriformes during evolution.

## Abstract (ru)

Курообразные (Galliformes) и гусеобразные (Anseriformes) – две ветви группы Galloanserae, базаль- ные по отношению к остальным Neognathae. В сравнении с Galliformes, эволюция кариотипов Anseriformes недостаточно изучена. Настоящее исследование посвящено представителям гусеобразных и изменению их хромосомных наборов в ходе эволюции. Была получена подробная информация о кариотипах (G-, C-бэндинг, флуоресцентная гибридизация in situ) трех видов уток: шилохвости (Anas acuta, 2n = 80), кряквы (Anas platyrhynchos, 2n = 80) и обыкновенного гоголя (Bucephala clangula, 2n = 80). С использованием зондов, разработанных на основе сортированных хромосом авдотки (Burhinus oedicnemus, 2n = 42, Charadriiformes), были выявлены районы гомологии на макрохромосомах и части микрохромосом уток. Изученные виды рода Anas имеют одинаковое число макрохромосом, при этом у B. clangula число крупных хромосом увеличено за счет двух разрывов предковых элементов. В отличие от представителей Anas, у этого вида обнаружены массивные гетерохроматиновые блоки в большинстве крупных макроаутосом и в половых хромосомах. Данные хромосомного пэйнтинга дополнены информацией о локализации рибосомной ДНК и амплификации теломерных повторов. Сравнительный анализ геномов с помощью приложения D-GENIES подтвердил высокий уровень консерватизма синтенных групп у Anatidae. Полученные результаты расширили представление о преобразованиях макро- и половых хромосом Anseriformes в ходе эволюции

## Introduction

Aves is one of the most specialized and species-rich groups of amniotes. Avian genomes are characterized by a relatively small size (1.4 Gb on average) (Gregory, 2023), a high degree of conserved synteny, and a relatively small proportion of repetitive DNA (Delany et al., 2000; Ellegren, 2013; Campagna, Toews, 2022). Birds’ diploid chromosome number (2n) varies from 40 to 142 owing to the presence of numerous microchromosomes (Christidis, 1990; Griffin et al., 2007; Degrandi et al., 2020). Despite the growing popularity of birds as a genomic research object, many species remain understudied both at the genomic and cytogenetic level. According to the Bird Chromosome Database, only 10 % of the species have a described karyotype, and comparative genomic studies have covered no more than 1 % of the species (Degrandi et al., 2020).

The order Anseriformes (geese, ducks, swans, and others) includes ~180 species (Gill et al., 2023) and together with Galliformes (chickens, guinea fowl, pheasants, and others) forms the Galloanserae clade. Galloanserae taxon diverged from other Neognathae approximately 100–70 million years ago (Van Tuinen, Hedges, 2001; Sun et al., 2017). Karyotypes of 46 Anseriformes species have been described. The diploid number of chromosomes varies from 2n = 74 (Spatula querquedula) (Ebied et al., 2005) to 2n = 98 (Coscoroba coscoroba) (Rodrigues et al., 2014). Nonetheless, most karyotypes contain 2n = 80–82 ((Bird Chromosome Database and (Degrandi et al., 2020)).

Eight Anseriformes species have been studied by comparative chromosome painting using chicken macrochromosome probes (Degrandi et al., 2020). The chicken (Gallus gallus, GGA, 2n = 78) genome is widely used as a reference in comparative genomics, and the painting probes have become essential for avian comparative cytogenetic researches (Shetty et al., 1999; Guttenbach et al., 2003; Griffin et al., 2008; Nanda et al., 2011; Islam et al., 2014; Rodrigues et al., 2014; Uno et al., 2019). Based on comparative data obtained using chicken painting probes, a hypothesis about homology of the first 10 macrochromosomes and Z chromosome in the avian ancestral karyotype has been propounded (Shibusawa et al., 2004; Griffin et al., 2007). Integration of microchromosomes into comparative cytogenetics can be implemented using microdissection (Zimmer et al., 1997; Griffin et al., 1999; Guillier-Gencik et al., 1999; Grützner et al., 2001; Masabanda et al., 2004), gene-specific probes (Coullin et al., 2005; Islam et al., 2014), and bacterial artificial chromosomes (BACs) (Shibusawa et al., 2001, 2002; Schmid et al., 2005; Fillon et al., 2007; Damas et al., 2017; Kretschmer et al., 2021). The set of avian probes obtained using chromosome sorting usually does not cover microchromosomes (Habermann et al., 2001).

The use of stone curlew (Burhinus oedicnemus; BOE) painting probes helps to include microchromosomes into cytogenetic analysis and to count linkage groups. The stone curlew has a karyotype with a diploid number 2n = 42 and contains only four pairs of microchromosomes (Bulatova et al., 1971). BOE painting probes have been applied to fluorescence in situ hybridization (FISH) experiments on a wide range of avian karyotypes, including reciprocal chromosome painting between the stone curlew and chicken (Hansmann et al., 2009; Nie et al., 2009, 2015; Wang et al., 2022). On the other hand, this type of analysis has not fully resolved the correspondence between microchromosomes (Nie et al., 2009). These data obtained using cytogenetic approaches and chromosome level genome assembly allow for the inclusion of microchromosomes into reconstructions of the avian ancestral karyotype (Damas et al., 2018).

There are several papers on Anseriformes chromosome evolution. In general, bird karyotypes have a low level of interchromosomal rearrangements (Griffin et al., 2008; Damas et al., 2018) and at the same time a high average rate of intrachromosomal rearrangements (Damas et al., 2019; O’Connor et al., 2024). Chicken BAC probes mapped onto Anas platyrhynchos chromosomes have revealed the presence of inversions in all macrochromosomes and in the Z chromosome of the duck relative to the chicken (Fillon et al., 2007; Kiazim et al., 2021). However, conservation of gene order among several Anseriformes species has been detected by means of gene- specific probes (Islam et al., 2014). Data on chromosome homology and rearrangements can be obtained by chromosomelevel assembly analysis (for example (Cabanettes, Klopp, 2018)). Now chromosome-level genome assemblies are available for 26 Anseriformes species in the NCBI database.

In the present work, we analyzed karyotype evolution of Anseriformes species by combining new data and previously published ones. New comprehensive cytogenetic results were obtained for three Anatidae species: the mallard (A. platyrhynchos, APL), the northern pintail (Anas acuta, AAC), and the common goldeneye (Bucephala clangula, BCL). Routine and differential staining (G- and C-banding) and molecular cytogenetic approaches (localization of ribosomal and telomeric painting probes and of the set of B. oedicnemus wholechromosome painting probes) were employed, and the results were integrated with previously obtained findings.

## Materials and methods

Sampled species and an ethical statement. Lung necropsy was performed on females of A. platyrhynchos, A. acuta, and B. clangula during the official hunting season in Novosibirsk Oblast (2009, 2008, 2023 years, respectively). For our research, we used one individual of each species. All experiments were approved by the Ethics Committee on Animal and Human Research at the Institute of Molecular and Cellular Biology (IMCB), the Siberian Branch of the Russian Academy of Sciences (SB RAS), Russia (decision No. 1/22 of 29 December 2022), following all relevant guidelines and regulations. This article does not contain any experiments on human subjects by the authors. The study was completed using equipment and materials of large-scale research facilities of the Cryobank of Cell Cultures, IMCB, SB RAS (Novosibirsk, Russia).

Primary fibroblast culture, metaphase chromosome preparation, and chromosome staining. Primary cell cultures were derived from lung necropsy as described previously (Romanenko et al., 2015). Metaphase spreads from all species were prepared from primary fibroblast cultures following standard procedures, including colcemid treatment, incubation in a hypotonic solution, and fixation in a methanol:acetic acid (3:1) mixture (Graphodatsky et al., 2001).

Routine Giemsa and/or DAPI staining was performed to identify morphology and count quantity of chromosomes. G- banding was done (Seabright, 1971) before FISH procedures. C-banding was performed for B. clangula and A. acuta according to a standard protocol (Sumner, 1972). Besides, for B. clangula, C-banding was done with modifications: metaphase chromosomes after Ba(OH)2 treatments were stained overnight with Chromomycin A3 (20 μg/ml) and 4′,6-diamidino- 2-phenylindole (DAPI; 50 ng/ml) and mounted in an antifade solution containing 1,4-diazabicyclo [2.2.2] octane (DABCO) (20 mg/ml) and 100 mM Tris-HCl pH 7.5 in glycerol.

Probe preparation, FISH, and microscopy. A set of whole-chromosome female DNA libraries (created from flowsorted chromosomes by degenerate-oligonucleotide-primed (DOP)-PCR (Telenius et al., 1992)) of the stone curlew was provided by Cambridge Resource Center for Comparative Genomics (Cambridge University, UK). The characterization of the set was described previously (Nie et al., 2009). All DNA libraries were labeled with biotin-11-dUTP and digoxigenin- 11-dUTP (Sigma) during secondary DOP-PCR amplification (Telenius et al., 1992) to create painting probes. A telomeric DNA probe was generated by PCR with oligonucleotides (TTAGGG)5 and (CCCTAA)5 (Ijdo et al., 1991). A ribosomal DNA probe was obtained from plasmid DNA (pHr13) containing a human partial 18S ribosomal RNA (rRNA) gene, a full human 5.8S rRNA gene, a partial 28S rRNA gene, and two internal spacers (Maden et al., 1987). Labeling was performed using the FTP-Display DNA Fragmentation Kit (DNA-Display, Russia) with the addition of 0.05 mM biotin- 11-dUTP or digoxigenin-11-dUTP (Sigma).

FISH was carried out by standard protocols (Graphodatsky et al., 2000; Liehr et al., 2017). For suppression of non-specific hybridization of probes in FISH experiments, chicken C₀t10 DNA was used. Digoxigenin-labeled probes were detected with the help of anti-digoxigenin-CyTM3 (Jackson Immunoresearch), whereas biotin-labeled probes were identified via sequential use of avidin-FITC (Vector Laboratories) and antiavidin FITC (Vector Laboratories). Images were captured and processed using a VideoTesT 2.0 Image Analysis System and a Baumer Optronics CCD Camera mounted on an Olympus BX53 microscope (Olympus).

Bioinformatic analysis. To perform the analysis of chromosome- level assemblies, we used the available material from GenBank NCBI. Chromosome-level assemblies of different Anseriformies species from different genera were compared with the A. platyrhynchos reference genome (GCA_008746955.3; 1n = 40, Z, W): A. acuta (GCA_963932015.1; 1n = 37, Z, W); Aythya baeri (GCA_026413565.1; 1n = 34, Z); Netta rufina (GCA_964035555.1; 1n = 40, Z, W); Cairina moschata (GCA_018104995.1, 29, Z); Clangula hyemalis (GCA_963989345.1; 1n = 40, Z); Anser cygnoides (GCA_026259575.1; 1n = 39); Cygnus olor (GCF_009769625.2; 1n = 34, Z, W); Oxyura jamaicensis (GCF_011077185.1; 1n = 33, Z, W). Dot plot comparative analysis was performed using the web application D-GENIES (Cabanettes, Klopp, 2018) by aligner Minimap2 v2.24.

## Results

Karyotypes of the three duck species

Кaryotypes of the three duck species were available: A. platyrhynchos (APL), A. acuta (AAC), and B. clangula (BCL) (Takagi, Makino, 1966; Beçak et al., 1973; Islam et al., 2014). The diploid chromosome number of all three species is 2n = 80. For each individual of the studied duck species, we counted the number of chromosomes in 30 routine stained metaphase plates. The A. platyrhynchos (mallard) karyotype was G-, R-, and C-banded before (Wójcik, Smalec, 2007) and the patterns coincided with our study.

**Fig. 1.**
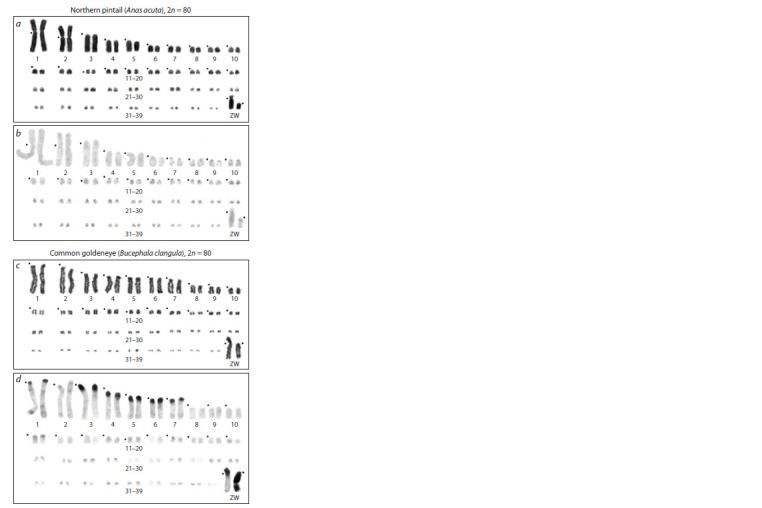
Routinely Giemsa-stained chromosomes of (a) A.acuta and (c) B. clangula and C-banded karyotypes of (b) A. acuta and (d) B. clangula Each black dot indicates a centromere position.

Cytogenetic data on A. acuta and B. clangula have been limited to Giemsa-stained karyotypes (Beçak et al., 1973; Abu-Almaaty et al., 2019). We performed Giemsa staining and C-banding for individuals of both species. The A. acuta karyotype was found to contain five pairs of large macro-autosomes (submetacentric pairs 1 and 2, and acrocentric pairs 3–5). The Z chromosome is a medium-sized subtelocentric macrochromosome, and the W chromosome is a small acrocentric chromosome (Fig. 1a). C-heterochromatin proved to be dot-like blocks in centromeric regions of almost all chromosomes except for large macroautosomes 1–5 and some microchromosomes (Fig. 1b). In the W chromosome, a pericentromeric heterochromatic block was observed.

B. clangula large macrochromosomes 1–7 and the W chromosome are acrocentric, whereas the Z chromosome is subtelocentric (Fig. 1c). Constitutive heterochromatin in this species was found to be localized in pericentromeric regions Fig. 1. Routinely Giemsa-stained chromosomes of (a) A.acuta and (c) B. clangula and C-banded karyotypes of (b) A. acuta and (d) B. clangula. Each black dot indicates a centromere position. of the six macroautosomes and the Z chromosome. In addition, the W chromosome is almost entirely heterochromatic (Fig. 1d). Overall, the B. clangula karyotype, as compared to the two Anas karyotypes, is enriched with repeated DNA organized in constitutive heterochromatin.

Localization of ribosomal genes and telomere repeats

Sequences carrying ribosomal genes and clusters of repeats homologous to rDNA were located on four small microchromosome pairs in A. acuta and A. platyrhynchos (Islam et al., 2014) and on two pairs in B. clangula (Supplementary Material 1)1.


Supplementary Materials are available in the online version of the paper: https://vavilovj-icg.ru/download/pict-2024-28/appx26.pdf


**Fig. 3.**
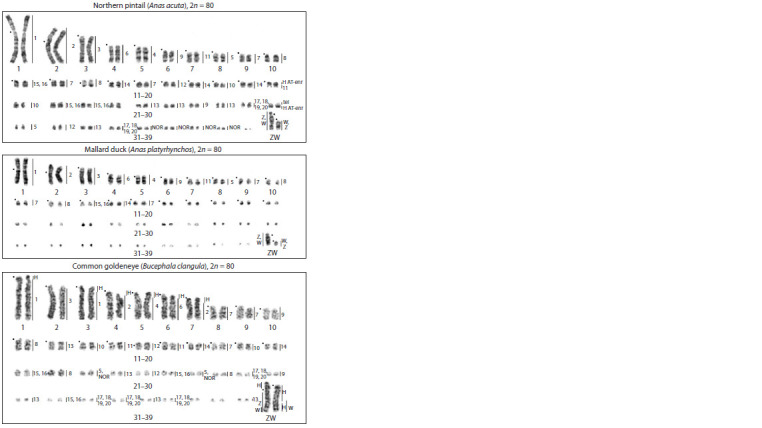
G-banded karyotypes of A. acuta, A. platyrhynchos, and B. clangula with assignment of homologies to B. oedicnemus chromosomes. ID numbers on the right indicate names of homologous stone curlew chromosomes; H – blocks of heterochromatin; NOR – positions of ribosomal DNA clusters; black circles indicate centromere positions; tel – a cluster of telomeric repeated sequences on a microchromosome containing a large DAPI-positive heterochromatic region in A. acuta.

In the A. acuta and B. clangula karyotypes, the probe containing (5′-TTAGGG-3′)n sequences detected small signals at the ends of all chromosomes except four or five pairs of dot-like microchromosomes with prominent signals. Besides, A. acuta was found to have a pair of microchromosomes where half of each chromosome was stained with a telomeric probe, and the other half contained a bright DAPI-positive region (AT rich) (Fig. 3 and Supplementary Material 1). We could not detect interstitial telomeric sites in macrochromosomes of the analyzed species.

Stone curlew homologies

**Fig. 2.**
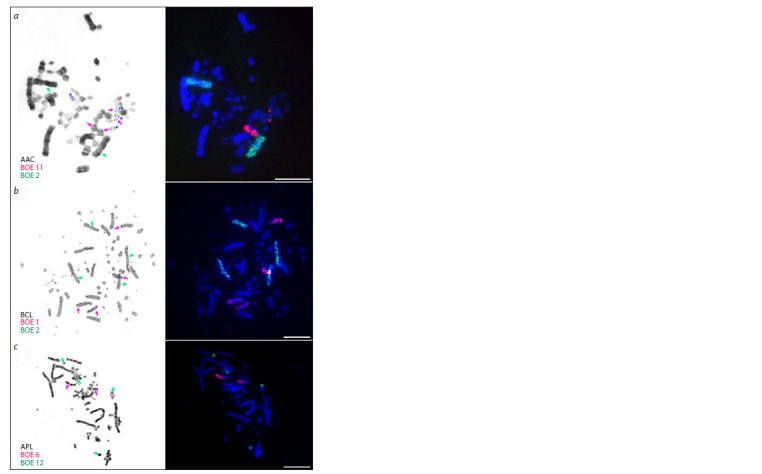
Examples of Burhinus oedicnemus (BOE) painting probes’ localization on karyotypes of Anatidae representatives (right) with GTG-banding of the same metaphase (left). a – hybridization of BOE 11 (red) and 2 (green) probes to A. acuta (AAC) chromosomes; b – hybridization of BOE 1 (red) and 2 (green) to A. platyrhynchos mallard (APL) chromosomes; c – hybridization of BOE 6 (red) and 12 (green) probes to B.clangula (BCL) chromosomes. Blue marks and * indicate microchromosomes carrying a large DAPI-positive heterochromatic region in A. acuta. Scale bars = 10 μm.

The complete set of BOE painting probes was hybridized to chromosomes of A. acuta, A. platyrhynchos, and B. clangula (Fig. 2). Owing to G-banding prior to FISH experiments, painted macrochromosomes were easily identified. We obtained specific signals on macrochromosomes and some microchromosomes of the three species. The probe containing the W chromosome also hybridized with Z: in A. acuta and A. platyrhynchos, weak signals along the chromosome body were detected; in B. clangula, a bright signal was detected in the subtelomere region of the q arm (Fig. 3). Also, the W chromosome signals on the B. clangula W chromosome covered subtelomere C-pozitive block. Other heterochromatic regions were not hybridized with BOE painting probes on B. clangula chromosomes. Signals on some microchromosomes were so weak that we failed to identify them. Quantities of microchromosomes covered by signals are presented in Table. Examples of some FISH results are shown in Fig. 2.

**Table 1.**
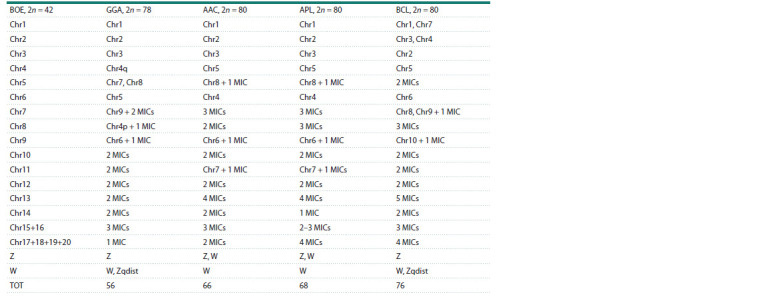
Chromosome correspondence between B. oedicnemus (BOE) and four Galloanseres species obtained using chromosome painting: Gallus gallus (GGA), A. acuta (AAC), A. platyrhynchos (APL), and B. clangula (BCL) Data on homology between GGA and BOE chromosomes are from (Nie et al., 2009). MICs are quantities of microchromosome pairs; Chr is the chromosome; TOT – the total quantities of chromosomes labeled by BOE painting probes; dist – the distal part of the chromosome.

Based on these results, comparative chromosome maps were created showing the homology between BOE and the three Anatidae species (see the Table, Fig. 3). Identification of many microchromosomes was limited by the resolution of the FISH method. Nonetheless, these maps gave an idea of relative sizes of labeled microchromosomes within size groups (pairs 11–20, 21–30, or 31–39). In total, homologies were identified for 66 out of 80 chromosomes of A. acuta, 68 out of 80 A. platyrhynchos chromosomes, and 76 out of 80 B. clangula chromosomes because each species has a different number of painted microchromosomes

## Discussion

Microchromosomes are typical of most amniotes (snakes, turtles, and lizards) with mammals and crocodilians being exceptions (Srikulnath et al., 2021; O’Connor et al., 2024). The presence of microchromosomes in avian karyotypes may account for the lag of bird cytogenetic studies behind mammalian studies. To date, 131 bird species from 47 families have been analyzed by comparative chromosome painting, i. e., no more than 2 % of extant avian species (Degrandi et al., 2020) (https://sites.unipampa.edu.br/birdchromosomedatabase).

The studied karyotypes of ducks from genera Anas and Bucephala possess identical diploid chromosome numbers (2n = 80). The diploid chromosome number reported for A. acuta varies among publications from 80 (Abu-Almaaty et al., 2019) to 82 (Beçak et al., 1973). A similar situation is true for B. clangula: diploid chromosome number 2n = 80 was shown for B. clangula americana (Beçak et al., 1973) and 2n = 84 for B. clangula (Hammar, 1970). Our results match the data obtained by A.H. Abu-Almaaty with co-authors and M.L. Beçak with co-authors, respectively: diploid chromosome number is 2n = 80 for both A. acuta and B. clangula (Fig. 1).

Previously it was reported that, even at the macrochromosomal level, chromosome banding can be difficult to identify, thereby making necessary a robust analysis of crossspecies homology (O’Connor et al., 2024). In this work we provided information about G-banding for three Anatidae species. This allowed reliable identification of all macro- and some microchromosome pairs. We used chromosome painting to gain a better understanding of the transformations that have caused the karyotype differences. Previously, comparative chromosome painting in Anatidae species has been performed only with chicken macrochromosome probes (Guttenbach et al., 2003; Nanda et al., 2011; Islam et al., 2014; Rodrigues et al., 2014; Uno et al., 2019), but Anseriformes karyotypes have not been investigated using BOE painting probes. Applying the set of BOE painting probes, we revealed homologies between B. oedicnemus, A. acuta, A. platyrhynchos, and B. clangula chromosomes. According to previously published findings (Nie et al., 2009), we added homologies of Gallus gallus (GGA) to our comparative analysis (see the Table)

Due to availability of chromosome-level genome assemblies of A. platyrhynchos and A. acuta, we prepared a dot plot analysis for genomes of Anseriformies species from different genera (Supplementary Material 3). A. platyrhynchos is a domestic species and its genome has been studied in detail and is usually used as the reference for this taxon. We made pairwise comparative analysis with A. platyrhynchos and eight genomes: A. acuta, Aythya baeri, Netta rufina, Cairina moschata, Clangula hyemalis, Anser cygnoides, Cygnus olor, and Oxyura jamaicensis. Karyotypes of most compared species contain 80 chromosomes, except for A. cygnoides and N. rufina (2n = 82) (Degrandi et al., 2020). The karyotype of C. hyemalis was not described. In almost all assemblies, the number of chromosome scaffolds is lower than haploid chromosome number and does not cover the smallest microchromosomes. The homologies between A. platyrhynchos and A. acuta, identified using chromosomal painting and dot plot analysis are consistent with each other (Supplementary Material 3a). The chromosome correspondence can also be traced at the G-banding level.

Macroautosome evolution in Anseriformes

**Fig. 4.**
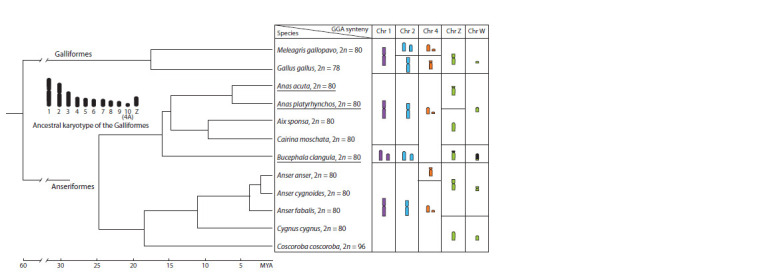
Schematic representation of chromosome rearrangements that occurred in macro- and sex chromosomes during the evolution of Galloanseres according to molecular phylogenies (Prum et al., 2015; Sun et al., 2017). Rearrangements of chromosomes that were identified by comparative painting with chicken probes (Guttenbach et al., 2003; Nanda et al., 2011; Islam et al., 2014; Rodrigues et al., 2014; Uno et al., 2019) or stone curlew probes (data obtained in this study, species names are underlined) are presented to the right of the molecular phylogeny. The ancestral karyotype of Galliformes and data about Galliformes species are from (Shibusawa et al., 2004; Griffin et al., 2007; Damas et al., 2019).

The most notable difference in macrochromosome structure among the three species is the presence of massive heterochromatic blocks in B. clangula macrochromosomes 1 and 3–7 (Fig. 1d). FISH with the stone curlew painting probes and dot plot analysis revealed conserved macrochromosome homologies between A. acuta and A. platyrhynchos. The diploid number (2n = 80) is typical for most Anatidae species, but in the B. clangula karyotype, we found an increase in the number of large chromosomes without a change in the diploid chromosome number. The reason is fission of ancestral chromosomes corresponding to GGA 1 and GGA 2 (Fig. 3, 4). A break in the centromere of ancestral chromosomes homologous to GGA 2 has been documented in other representatives of Galloanseres, for example, in the turkey (Shibusawa et al., 2004). In other avian lineages, there are independent fissions of orthologs of GGA 2 around the centromere (Degrandi et al., 2020). The ancestral homolog of GGA chromosome 1 was split in two within Passeriformes’ ancestral karyotype (Damas et al., 2018).

Orthologous elements of GGA 4 are represented by one macrochromosome and one microchromosome in the three examined ducks (see the Table). By contrast, the GGA 4 probe hybridized to one macrochromosome in Anser anser (Fig. 4) (Gut-tenbach et al., 2003). Ancestral chromosome 4 (corresponding to GGA 4q) is the most ancient of all the avian chromosomes, appearing intact even in humans (Chowdhary, Raudsepp, 2000). In the GGA genome, we can see the result of fusion of avian ancestral chromosomes 4 and 10 (4A) (GGA 4q and 4p) (Shibusawa et al., 2002; Guttenbach et al., 2003; Derjusheva et al., 2004; Itoh, Arnold, 2005; Griffin et al., 2007; Damas et al., 2018), and 4p still retains properties of a microchromosome (high gene density and recombination rate, CpG island distribution). The observed interchromosomal changes have also taken place in different lineages (Fig. 4) and led to genome reorganization in the extant species. These observations may be best explained by rearrangements with common breakpoint reuse.

The data of dot plot analysis also identified the presence of interchromosomal rearrangements affecting macrochromosomes in C. moschata and O. jamaicensis (Supplementary Material 3d, h). In C. moschata, fission of chromosomes orthologous to A. platyrhynchos 6 and 7 and fusion parts of these chromosomes with chromosomes 5 and 8 respectively are observed (Supplementary Material 3d). Identified rearrangements are not confirmed using chromosome painting (Islam et al., 2014). This may be due to either a weak signal that would not be detected by chicken chromosome painting probes, or an artifact of de novo genome assembly. Also, in O. jamaicensis, genome fusion of chromosomes orthologous to A. platyrhynchos 4 and 11 was detected (Supplementary Material 3h). Minor interchromosomal rearrangements in pericentromeric regions of almost all macrochromosomes were observed.

Evolution of microchromosomes in Anseriformes

Bird microchromosomes have attracted research attention because although for most bird species these chromosomes represent 25 % of the genome, they are known to encode 50 % of all genes (Smith et al., 2000). It has been hypothesized that avian microchromosomes represent archaic linkage groups of ancestral vertebrates and have been preserved in a conserved state throughout the evolution of birds (Burt, 2002; Nakatani et al., 2007). Among Anatidae species, only A. platyrhynchos microchromosomes have been examined by FISH with chicken BAC probes, and the obtained data have confirmed the idea of high conservatism of avian genomes (Fillon et al., 2007; Kiazim et al., 2021).

The set of BOE painting probes covered not all duck microchromosomes (see the Table, Fig. 3). Enrichment of some microchromosomes with ribosomal DNA sequences and telomeric repeats may also influence the detection of homologous sequences on microchromosomes. Sizes of homologous segments on microchromosomes may be below the resolution level of molecular cytogenetics. This problem may be exemplified by BOE 5 and orthologous elements in chicken and duck karyotypes. This probe delineated GGA 7 and GGA 8 but only two pairs of small microchromosomes in B. clangula. Most likely, BOE 5 labels more microchromosomes in ducks, and some signals were overlooked when the FISH data were inspected.

Although all three ducks examined here have the same diploid number, in the B. clangula chromosomal set, we suggest there are at least two ancestral macrochromosome fissions. It is possible that some microchromosomes have merged or fused with macrochromosomes during the formation of the B. clangula karyotype. For Galloanserae, interchromosomal rearrangements between microchromosomes have not yet been recorded, but for other orders of Neognathae, data on interchromosomal rearrangements involving macro- and microchromosomes and the fusion of microchromosomes are gradually accumulating (reviewed in (O’Connor et al., 2024)). In addition, many researchers believe that the ancestral element 10 (4A) is a microchromosome according to its GC content and gene density (Griffin et al., 2007; Damas et al., 2018; O’Connor et al., 2024). Fusion of ancestral elements 10 (4A) with 4 is often observed in Anseriformes (Fig. 4).

Another example of the fusion of micro- and macrochromosomes was revealed by dot plot analysis in Galloanserae species O. jamaicensis (Supplementary Material 3h). Fusions of microchromosomes occurred in birds; for example, numerous fusions of microchromosomes took place during formation of the stone curlew karyotype (Nie et al., 2009). The fusion of microchromosomes was revealed in the genome of C. moschata (Supplementary Material 3d). The use of BAC clones to analyze the chromosome set of B. clangula and chromosome-level genome assembly will help to identify rearrangements that occurred during the formation of the karyotype of this species

Given the low number of repeated sequences in bird genomes, the large number of telomeric sequences (2 to 4 %) is a point of interest (Delany et al., 2000). In addition to the canonical localization of (5′-TTAGGG-3′)n sequences at the ends of chromosomes, these highly conserved sequences may be situated at an interstitial position: around a centromere or inside a chromosome arm. Three types of telomere arrays differing in length, position on a chromosome, age-related stability, and linked either to macro- or to microchromosomes have been described in the chicken (Delany et al., 2000, 2007; Rodrigue et al., 2005). It has been demonstrated that some avian microchromosomes tend to accumulate telomeric repeats. So-called mega-telomeres (200 kbp to 3 Mbp) at one of the ends have been detected in three chicken chromosomes from highly inbred White Leghorn line (9 and two microchromosomes) (Rodrigue et al., 2005; Delany et al., 2007).

Similar features of the distribution and amplification of telomeric sequences have been described for representatives of Passeriformes (Dos Santos et al., 2015, 2017) and three anserid species (Islam et al., 2014). In the C. moschata karyotype, 14 pairs of microchromosomes bear extended telomeres (Nanda et al., 2002). We also located extended telomeric sequences at the ends of four microchromosome pairs in B. clangula and 4–5 dot-like microchromosome pairs in A. acuta (Supplementary Material 1). Further studies involving larger number of individuals, mapping BAC probes to identify individual chromosomes, are needed to determine whether microchromosomes with large clusters of telomeric repeats are common to all Galloanserae, or whether different microchromosomes tend to accumulate telomeric sequences in different bird species.

Genes of 18S–28S rDNA are believed to have been located on a single pair of microchromosomes in the ancestral avian karyotype, as observed in palaeognathous birds and in the chicken (Nishida-Umehara et al., 2007; Delany et al., 2009). We found clusters of rRNA genes on four microchromosome pairs in A. acuta and two pairs in B. clangula. Localization of rDNA sequences on several pairs of microchromosomes was also described for A. platyrhynchos, C. moschata, and Anser cygnoides (Islam et al., 2014).

Some bird microchromosomes have accumulated different types of repeated sequences during karyotype evolution (Nanda et al., 2002; Dos Santos et al., 2015, 2017; Zlotina et al., 2019; de Oliveira et al., 2024). We revealed the presence of microchromosomes enriched with telomeric sequences and/or constitutive heterochromatin in A. acuta (Fig. 3 and Supplementary Material 1). The data presented here are based on the analysis of one individual from each investigated species. Perhaps accumulation of repetitive sequences is subject to intraspecies variation, and we need more information to address this issue.

Sex chromosome evolution

There is variation in morphology of Z chromosomes of Galloanseres in terms of centromere position and size of heterochromatin regions (Fig. 3). For example, it was reported previously that the centromere position on the Z chromosome is different between the chicken and turkey, but most likely this situation is a consequence of the accumulation of heterochromatin on the chicken Z chromosome (Shibusawa et al., 2004). In Anseriformes, submetacentric, subtelocentric, and acrocentric Z chromosomes were observed (Fig. 4). Recent research indicates that the bird Z chromosome is involved in inter- and intrachromosomal rearrangements (Damas et al., 2019). Dot plot analysis of eight Anseriformies species identified the same gene order on the Z chromosome in all species except for Anser cygnoides (Supplementary Material 3f ). Different chromosome morphology (Fig. 4) with gene order conservation (Supplementary Material 3) indicates the presence of centromere repositions in Anseriformes Z chromosomes. Amplification of heterochromatin (AT- and GC-rich) could occur in the pericentromeric region of the Z chromosome of B. clangula (Fig. 1 and Supplementary Material 2).

Physical size of the W chromosome is one of highly variable characteristics of the avian genome (Stiglec et al., 2007). Usually, the W chromosome of Neognathae is smaller than the Z chromosome and has lost most genes. Nonetheless, large W chromosomes in many avian species have been described (Rutkowska et al., 2012; De Oliveira et al., 2017). The size of the degenerated homologous sex chromosomes may increase due to the enrichment with repetitive elements or to autosomal translocations (Pala et al., 2012; Schartl et al., 2016). Among the three species under study, a large W chromosome has been previously described only for B. clangula (Hammar, 1970; Beçak et al., 1973). C-banding of B. clangula metaphase chromosomes revealed that its W chromosome carries extended heterochromatic regions with a prominent AT-rich interstitial region (DAPI-positive) and GC-rich subtelomeric and pericentromeric regions (Chromomycin A3–positive) (Supplementary Material 2).

It is interesting that AT- and GC- heterochromatic regions overlapped. It may be explained by alternation of AT- and GC-rich repeats or the presence of repeats with a uniform distribution of AT/GC nucleotides in these regions. The painting probe containing the W chromosome covered the euchromatic region and subtelomere C-positive block, which may indicate the presence of some W-specific repeated sequences in the distal part of W. Remarkably, this probe hybridized also with the distal region of the q arm of the Z chromosome (Fig. 3). This may indicate a pseudoautosomal region or localization of amplified W-specific repeated sequences. Further investigation of B. clangula genomic architecture, repeatome, and whole-genome assembly may shed light on the evolutionary rearrangements of sex chromosomes.

## Conclusion

Here, for the first time, we used stone curlew painting probes to analyze karyotypes of three Anatidae representatives. The detailed comparative cytogenetic maps cover all macrochromosomes and some microchromosomes and contain valuable information about the composition of the karyotypes, chromosome morphology, and localization of some repeated DNA sequences. Data of comparative chromosome painting together with dot plot (D-GENIES) analysis confirm the established view about the high level of syntenic group conservation among duck genomes

Some microchromosomes in the examined duck karyotypes were found to bear amplified clusters of ribosomal DNA, telomeric repeats, or fractions of repeated DNA. The rDNA clusters were located on more than one pair of microchromosomes in Anatidae karyotypes – on four microchromosome pairs in A. acuta and two pairs in B. clangula. Also, microchromosomes with extended telomeres have been found in this work. This state of affairs may be a contributing factor to the difficulties with assembling avian genomes by conventional bioinformatic methods. Accumulation of heterochromatin in the B. clangula karyotype needs further research: karyotyping of more individuals and repeatome analysis.

## Conflict of interest

The authors declare no conflict of interest.
